# Rate perception adapts across the senses: evidence for a unified timing mechanism

**DOI:** 10.1038/srep08857

**Published:** 2015-03-09

**Authors:** Carmel A. Levitan, Yih-Hsin A. Ban, Noelle R. B. Stiles, Shinsuke Shimojo

**Affiliations:** 1 Cognitive Science, Occidental College, 1600 Campus Road, Los Angeles CA 90041; 2 Computation and Neural Systems, California Institute of Technology, 1200 E. California Blvd, Pasadena, CA 91125; 3 Division of Biology and Biological Engineering, California Institute of Technology, 1200 E. California Blvd, Pasadena, CA 91125

## Abstract

The brain constructs a representation of temporal properties of events, such as duration and frequency, but the underlying neural mechanisms are under debate. One open question is whether these mechanisms are unisensory or multisensory. Duration perception studies provide some evidence for a dissociation between auditory and visual timing mechanisms; however, we found active crossmodal interaction between audition and vision for rate perception, even when vision and audition were never stimulated together. After exposure to 5 Hz adaptors, people perceived subsequent test stimuli centered around 4 Hz to be slower, and the reverse after exposure to 3 Hz adaptors. This aftereffect occurred even when the adaptor and test were different modalities that were never presented together. When the discrepancy in rate between adaptor and test increased, the aftereffect was attenuated, indicating that the brain uses narrowly-tuned channels to process rate information. Our results indicate that human timing mechanisms for rate perception are not entirely segregated between modalities and have substantial implications for models of how the brain encodes temporal features. We propose a model of multisensory channels for rate perception, and consider the broader implications of such a model for how the brain encodes timing.

Timing is critical to neural processing of visual and auditory events. The brain processes complex temporal stimuli and can be sensitive even to small shifts in timing. The mechanisms underlying this feat are not well understood, but the metaphor of a clock provides a helpful way to consider potential models of time perception^1^. These models^2^ typically include a pacemaker and an accumulator, which could be central (and thus supramodal) or more local, and have been extensively applied to studies of duration perception. In principle, such models could also be used to extract rate information from the world. Alternate models posit that multiple distributed networks of neurons are involved in different temporal tasks^3^,^4^.

The fundamental question motivating these experiments is whether the brain uses modality-specific timing mechanisms or a unified timing mechanism. Previous rate perception experiments have shown that auditory information can influence perception of the rate of concurrently presented visual stimuli^5^,^6^,^7^,^8^; when participants are asked to judge visual flicker rate, auditory information biases their judgments. When auditory and visual reliability are matched, concurrent visual information can also influence perception of auditory rate and a simple Bayesian model of multisensory integration that uses reliability information is able to predict performance^8^. However, some studies of duration perception have suggested that audition and vision process duration separately^9^,^10^,^11^. These studies, which all used adaptation paradigms, found that changes in duration perception seem to be sensory-specific; perception of temporal properties in an unadapted modality was unchanged by adaptation. A channel-based model successfully described the pattern of duration adaptation within an adapted modality; with increasing discrepancies between adaptor and test duration, the magnitude of the aftereffect was reduced^11^. Another piece of evidence for modality-specific timing mechanisms comes from the result that visual, but not auditory, timing between stimuli is distorted around the time of eye movements^12^.

Previous studies of rate generally used concurrent stimulation to study crossmodal interactions^5^,^6^,^7^,^8^; thus the results of those studies can be explained either using a multisensory timing mechanism for rate or separate unisensory mechanisms that are part of a larger network for rate perception. Thus the primary aim of the current study was two-fold: (1) to examine whether processing of rate is unified and supramodal or whether it is modality-specific and (2) to use non-concurrent stimulation of audition and vision. We also (3) tested whether a channel-based model would apply to rate perception by varying the difference between our adaptation and test temporal frequencies.

## Results

**Figure 1:**
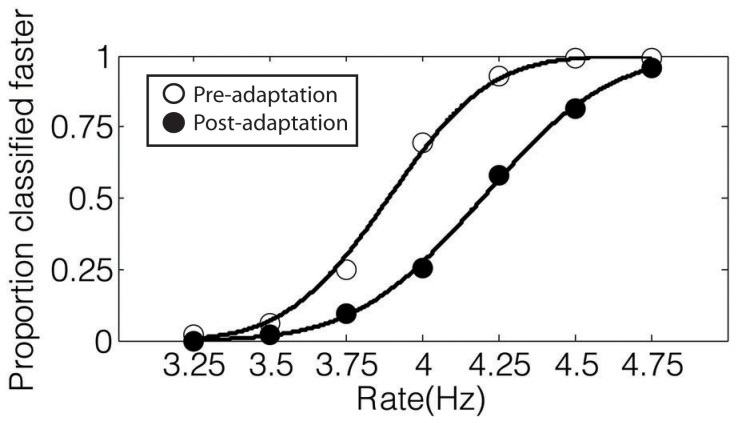
Calculation of aftereffects. The PSE was calculated for the pre- and post-adaptation test trials by fitting a cumulative normal function and finding the 50% point. The aftereffect was quantified as the difference (post – pre) in PSE. Above psychometric functions (data combined across all 8 participants) for the AA (auditory adapt, auditory test) “fast” condition of the main experiment are shown with open circles depicting pre-adaptation data and filled circles representing post-adaptation data.

Anonymized data for both experiments is available at http://figshare.com/articles/crossmodal_rate_perception_data/1310442. Data for each condition were analyzed by calculating the Point of Subjective Equality (PSE) at which participants were equally likely to classify a stimulus as fast or slow. All psychometric functions were fit in MATLAB (Mathworks, RRID: nlx_153890), using the Palamedes toolbox^13^. This toolbox allows for testing of whether specific parameter values are statistically significantly different across psychometric functions, using a Maximum Likelihood criterion. Because our participants received feedback on the pre-adaptation trials but not on the post-adaptation trials, a change in slope could reflect this difference or stem from the adaptation. Thus to test for effects of adaptation, we explicitly focused on PSE. We conducted three sorts of analyses of the data in our main experiment: (1) an ANOVA based on individual shifts in PSE, (2) binomial analyses based on whether individual shifts in PSE were in the predicted directions, and (3) a model comparison looking at shifts in the PSE calculated by combining together the raw data from individual participants into pooled psychometric functions. Figure 1 depicts the magnitude of the aftereffect as the difference between the PSE in the test trials presented before and after adaptation for the pooled data, and Supplemental Table 1 provides individual participant PSEs and slopes for every condition.

**Figure 2:**
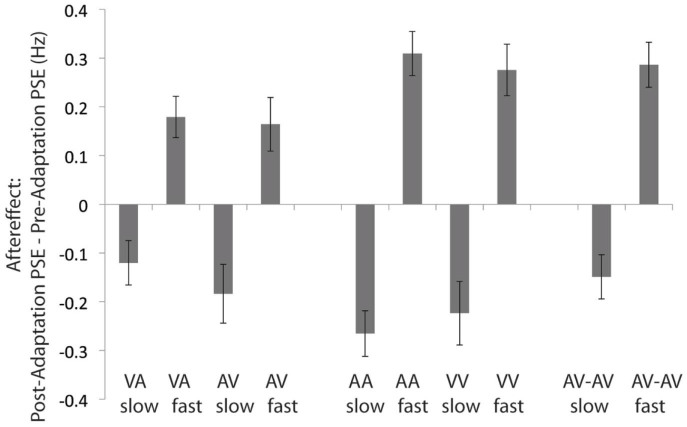
Crossmodal, unimodal, and bimodal aftereffects. Participants (*N* = 8) in the main experiment completed the rate classification task pre- and post-adaptation to relatively slow or fast stimuli for AV (auditory adaptor, visual test), VA (visual adaptor, auditory test), AA (auditory adaptor/test), VV (visual adaptor/test), and AV-AV (bimodal) conditions. The aftereffect was the post-adaptation PSE – pre-adaptation PSE. Error bars show 95% confidence intervals.

The most critical result of this paper is the finding that adaptation transfers across modalities, even when the modalities are never presented together. In our main experiment, participants experienced unimodal (test and adaptation in the same modality), crossmodal (test and adaptation in different modalities), and bimodal (test and adaptation both audiovisual) conditions. As shown in Figure 2, in all ten conditions, the PSE shifted in the direction of the adaptor, indicating a negative aftereffect. That is, after exposure to a fast adaptor, stimuli were perceived to be pulsing more slowly than prior to adaptation, and the reverse occurred after exposure to a slow adaptor. We conducted a 2×5 ANOVA with the independent variables of adaptor rate and of modality. The adaptor rate could be relatively fast or relatively slow, and modality could be AV (auditory adaptor, visual test), VA, AA, VV, and AV-AV (concurrent bimodal adaptor and test). We found a significant effect of adaptor rate, F(1,7) = 44.98, *p* < 0.0005. There was no significant effect of modality, F(4,4) = 0.19, *p* = 0.93 and there was no significant interaction between adaptor rate and modality, F(4,4) = 1.20, *p* = 0.43. Thus we demonstrated that adaptation influenced temporal rate perception. We also conducted binomial analyses in which we assessed whether each individual psychometric function shifted in the expected direction. With 8 participants and 10 conditions, this resulted in 80 potential shifts in PSE. Of those, 70 shifted in the expected direction. A binomial test indicated a result of 70 or more shifts in the expected direction was statistically significant, *p < 0.000001*. Examining just the crossmodal conditions, 26 of 32 shifts were in the expected direction, which the binomial test also demonstrated was statistically significant, *p < 0.0005*. Finally, we examined the pooled psychometric functions for each of the ten conditions by comparing two models: a constrained model in which the PSE was fixed to be the same value in both pre- and post-adaptation psychometric functions (but the slope was not fixed) and an unconstrained model in which the PSE and slope were both free to vary across the pre- and post-adaptation psychometric functions. Palamedes provides both simulated p-values and those based on theoretical chi-squared distributions based on sampling the empirical data. All statistical tests and confidence intervals were calculated using this approach, using 5000 iterations of the model. Here, we report the p-values derived from the chi-square analysis, but in all cases the p-values of both were very similar. For every condition, the model allowing for the pre- and post-adaptation psychometric functions to have a different PSE fit the data significantly better (two-tailed test, all *p* < 0.0001; note that a Bonferroni-corrected cut-off for significance would be *p* < 0.005 as 10 comparisons were made), demonstrating that adaptation influenced temporal rate perception in all conditions. Thus we concluded that the adaptation occurs in unimodal and bimodal conditions and also transfers across modalities in crossmodal conditions.

Using our pre-adaptation data, we calculated the reliability for auditory-only, visual-only, and bimodal conditions as the inverse of the measurement variance^14^. We grouped together all pre-adaptation conditions of a particular modality regardless of adaptation condition. These results were not conclusive (our experimental design was focused on PSE rather than reliability), but were inconsistent with the maximum likelihood estimation (MLE) model based on reliability^15^, which predicts that the bimodal reliability should be the sum of the unimodal reliabilities. Because audition was more reliable than vision for our task (see Supplemental Figure 1), if relative reliability predicted the magnitude of adaptation, we would expect more adaptation in the AV (auditory adaptation, visual test) condition than in the VA (visual adaptation, auditory test) condition, but this was not supported by our aftereffect data (Figure 2). The Supplemental Note has more details on these analyses and their implications.

**Figure 3:**
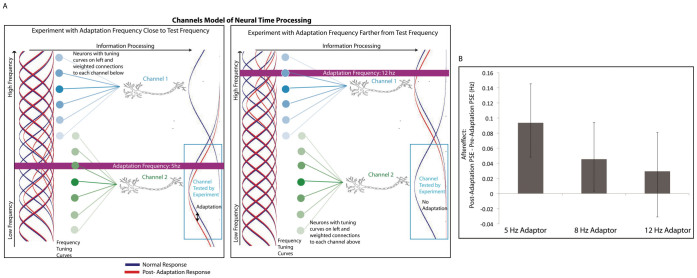
Aftereffect suggests rate perception channels. (A). A potential neural network mechanism of channel adaptation. Sensory neurons with frequency tuning curves (blue and green circles) make weighted connections (colored lines) to neurons in different channels. Each channel is sensitive to different frequency ranges. (B). Participants (*N* = 8) in the channels experiment completed the visual rate classification task pre- and post-adaptation to relatively fast auditory adaptation stimuli (5 Hz, 8 Hz, and 12 Hz in different sessions). The aftereffect was the shift in PSE. Error bars show 95% confidence intervals.

A physiologically plausible approach to our findings is to use a channel-based model in which groups of neurons are maximally sensitive to different temporal frequencies but have overlapping, narrow tuning curves. In such a framework, repeated presentation of the adaptation stimulus would lead to a reduction in response by the neurons sensitive to the frequency of the adaptor^11^,^16^. This shifts the overall response of a population of neurons away from the adaptor, creating a negative aftereffect, but only for a limited range of frequencies (Figure 3A). Our channels experiment tested whether the aftereffect was based on narrowly-tuned specific frequency channels or reflected a higher-level, non-specific (and possibly cognitive) effect. We probed this question by examining the effect of the difference in rate between the adaptor and the test in the crossmodal condition with an auditory adaptor that was faster than the visual test stimuli. We again tested the difference between models in which the PSE was constrained to be the same for pre- and post-adaptation and in which the PSE was unconstrained (all two-tailed tests). The model allowing for a difference in PSE fit the data significantly better for the 5 Hz condition (*p* = 0.0004), but not the 8 Hz (*p* = 0.07) or 12 Hz (*p* = 0.27) conditions. Thus, we found that the aftereffect was only significant when the discrepancy between adaptor and test frequencies was relatively small (Figure 3B), consistent with a model of narrowly-tuned channels for temporal frequency (Figure 3A). If our effect were due to response bias, a larger discrepancy between adaptor and test stimuli would likely induce a larger, or at least the same, aftereffect. We found the opposite pattern, suggesting that the rate aftereffect is, at least in part, occurring at the sensory level of temporal mechanisms.

## Discussion

Our results indicate vigorous and quick interactions between the senses even without concurrent stimulation. The transfer of adaptation across the senses suggests that timing mechanisms for rate perception cannot be solely unimodal, in contrast to previous studies of duration that have indicated modality-specific timing mechanisms^1^,^10^,^11^,^12^. Our results imply either a supramodal mechanism or strong linkages between modality-specific mechanisms for perception of rate. In the following, we will compare our results to those of related studies, consider possible methodological explanations for our data, and discuss the relationship of the current findings to classical and modern theories of multisensory integration and to the channels model of adaptation. We will then conclude with the neural implications of our work.

The magnitude of our aftereffect was similar to that induced by concurrent stimulation of vision and audition with discrepant rates^7^. In that study, participants experienced concurrent auditory and visual pulses at the same or different rates. The test task required participants to indicate whether the auditory and visual pulses were presented at the same rate and was performed before and after adaptation to synchronous or discrepant rates. For both slow and fast discrepant cases, exposure to the discrepancy shifted the point at which the auditory and visual rates were perceived to be the same; the aftereffect was approximately 0.2 Hz (relative to the 4.0 Hz baseline). During concurrent stimulation, one might expect crossmodal interactions. Indeed, it has been the focal motivation of multisensory research to understand the mechanisms underlying concurrent crossmodal interactions. Both psychophysical experiments and models have concentrated on situations with concurrent stimulation of the senses, often using an adaptation paradigm to look for aftereffects of consistent and inconsistent multisensory input. Thus, most researchers have not addressed the question of crossmodal changes when there is no concurrent multisensory stimulation, or even assumed that nothing crossmodal would occur. Crossmodal motion aftereffects^17^,^18^ and numerosity aftereffects^19^ have previously been shown as exceptional cases of nonconcurrent crossmodal changes, and our result demonstrates that such transfer of adaptation also applies to the perception of temporal frequency.

The specific methodological approach was crucial to uncovering this result. By using the method of single stimuli, we created an internalized criterion of perceived median rate for participants to use in judging relative rate of test stimuli. If participants were comparing the rate of post-adaptation trials only to that of the recent test stimuli, then no shift in PSE would be observed as any change in perceived rate of the test stimuli would lead to a corresponding change in the perceived median. We believe that the observed shifts in PSE occurred because adaptation changed the perceived rate of post-adaptation trials as judged relative to a criterion registered from pre-adaptation trials (*i.e.*, an adaptive change at perceptual levels). However, an alternate possibility is that adaptation shifted the internal criterion (or that it shifted both the internal criterion and the perceived rate of post-adaptation trials). Our channels experiment demonstrated that an effect only occurred when the rate of the adaptor was relatively similar to the rate of the test stimuli, which rules out response bias as the sole explanation for our results. The shifted criterion explanation may still be possible if one posits a model such that adaptation stimuli only influenced the internal criterion within a narrow window, which would imply that the internalized rate was itself multisensory. Either one of these interpretations (change in perception or change in criterion) implies some modality-nonspecific mechanism for perceiving or internalizing rate. Future experiments using a rate reproduction task could help disambiguate these possibilities.

Models based upon reliability may not be the appropriate approach for understanding the mechanisms the brain uses for adaptive change with non-concurrent stimulation. It is possible that reliability predicts the perceptual integration of conflicting concurrent stimuli, but not the recalibration resulting from repeated exposure to such stimuli^20^,^21^. Instead, accuracy of each sensory cue, as well as priors about the consistency of mapping between particular sensory cues, both likely determine the magnitude and rate of adaptation^15^ in those cue-conflict situations. However, in our experiments there is no sensory discrepancy to resolve. Thus, the crossmodal rate aftereffect may reflect a different type of adaptation from the sensory recalibration of two concurrent signals and an approach to modeling that does not directly rely on reliability or accuracy is necessary to understanding the result (see Supplemental Note for further discussion).

The channels model^16^ provides an approach to modeling adaptation based on considering neural mechanisms that could underlie adaptation. Consistent with the predictions of local repulsion due to repeated presentation of an adaptor, we found negative aftereffects in all of the conditions in our main experiment, and our channels experiment confirmed that the aftereffect is attenuated as the adaptation frequency becomes more different from the test frequencies. Channel models have been previously used to explain negative aftereffects for both auditory and visual duration perception^11^ and the auditory rhythm aftereffect^22^. In both of those cases, however, the channels were sensory-specific; adaptation did not transfer crossmodally. Our crossmodal results require either supramodal channels or a mechanism for vigorous interaction between sensory-specific channels without concurrent stimulation. With concurrent audiovisual stimulation, interference in rate discrimination only occurs when the auditory and visual rates are similar^7^,^8^ and the same is true with concurrent auditory and tactile stimulation^23^; these, together with the current findings, are consistent with the notion that there are tuned crossmodal channels for rate perception.

Although previous studies of duration perception that have used an adaptation paradigm have suggested a dissociation between audition and vision^9^,^10^,^11^, other recent studies complicate this simple picture. A single clock mechanism that is not modality-specific best explains distortions that arise in perception of duration of crossmodally-defined intervals^24^. A non-specific mechanism also is consistent with results showing that, under conditions of uncertainty, auditory and visual information about duration is integrated in a statistically optimal fashion^25^ and with the recent finding that training in auditory or tactile interval discrimination improves participants' ability to discriminate types of visual apparent motion^26^.

Our findings are consistent with the adaptive processing hypothesis that many, if not all, cortical areas are essentially multisensory and that differences in activation during particular tasks may depend on the particular demands of the required processing, rather than on the modality in which the information is transmitted^18^,^27^. It may be that rate is primarily encoded in the auditory cortex and that visual information about rate is converted to an auditory representation, perhaps functioning as a “supramodal clock,” in this case^28^,^29^, either via direct input from visual areas or through feedback from multisensory areas^30^. Separate neural substrates may underlie different sorts of timing tasks; within audition, duration-based timing activates an olivocerebellar network, while a striato-thalamo-cortical network is involved in beat-based timing^31^. Neural entrainment^32^ is a biological mechanism that could plausibly explain our findings at a neuronal level. Our rate perception aftereffect and the crossmodal motion aftereffects^17^,^18^ both involve processing of events that unfold over time. A timing mechanism that processes similar events across modalities would facilitate such processing. The current findings demonstrate a new approach to investigating crossmodal, adaptive changes at sensory/perceptual levels, when there is no concurrent stimulation.

## Methods

### Participants

Eight participants (5 females) took part in the main experiment. Eight participants (3 females) took part in the channels experiment; two of those participants were common to both studies. All participants gave informed written consent.

### Design

We conducted two experiments using this paradigm. Our main experiment explored whether rate adaptation would occur, and whether it would transfer in crossmodal conditions. All participants in the main experiment experienced ten conditions in pseudorandom order on separate days. Conditions in this experiment were “slow” (3 Hz) and “fast” (5 Hz) adaptors in AV (auditory adaptor, visual test), VA, AA, VV, and AV-AV (concurrent bimodal adaptor and test) sessions (details below). The channels experiment tested whether the crossmodal transfer would occur at larger discrepancies between adaptor and test temporal frequencies. Participants in that experiment experienced 5 Hz, 8 Hz, and 12 Hz AV (auditory adaptor, visual test) sessions on different days in pseudorandom order. The Caltech Committee for the Protection of Human Subjects approved the studies and the methods were carried out in accordance with the approved guidelines.

### Apparatus

We used customized equipment that controls timing with <2 ms precision. This consisted of a microcontroller that communicated with a box that had three LEDs mounted in front of a speaker; there was a red LED (radius 3.2 mm) aligned with the center of the speaker and two green LEDs 2.5 cm above and below the center. The red LED was used to present the visual stimuli. The speaker played a 440 Hz tone. Participants were seated approximately 60 cm away from the apparatus, and were unrestrained. Responses were via key press. The volume of the sounds at the head was approximately 55 dB. The room was totally dark except for the LEDs.

### Test Task

There were seven pulse rates ranging from 3.25–4.75 Hz (square wave with a 50% duty cycle), and the number of pulses in each sequence was randomized between 5 and 10 so that neither the number of pulses nor the total duration of the pulse train was a useful cue to rate. We used the method of single stimuli^33^, in which participants judged individual stimuli relative to the mean or median of a group of stimuli; this method has been used widely since it is simple yet reliable. We did not provide an explicit reference standard; instead, participants were exposed to training stimuli that included all of the test rates and were given feedback as they classified them as relatively fast or slow. After a minimum of 14 training trials, there were 140 pre-adaptation test trials (with feedback) on this fast/slow discrimination task. After the initial adaptation phase, test and adaptation blocks were alternated. There were a total of 140 post-adaptation test trials across 20 blocks; within each block of 7 trials, each pulse rate was presented and no feedback was given on these trials. Participants were explicitly instructed to judge the post-adaptation test trials in the same way that they had judged the pre-adaptation trials.

### Adaptation Task

**Figure 4:**
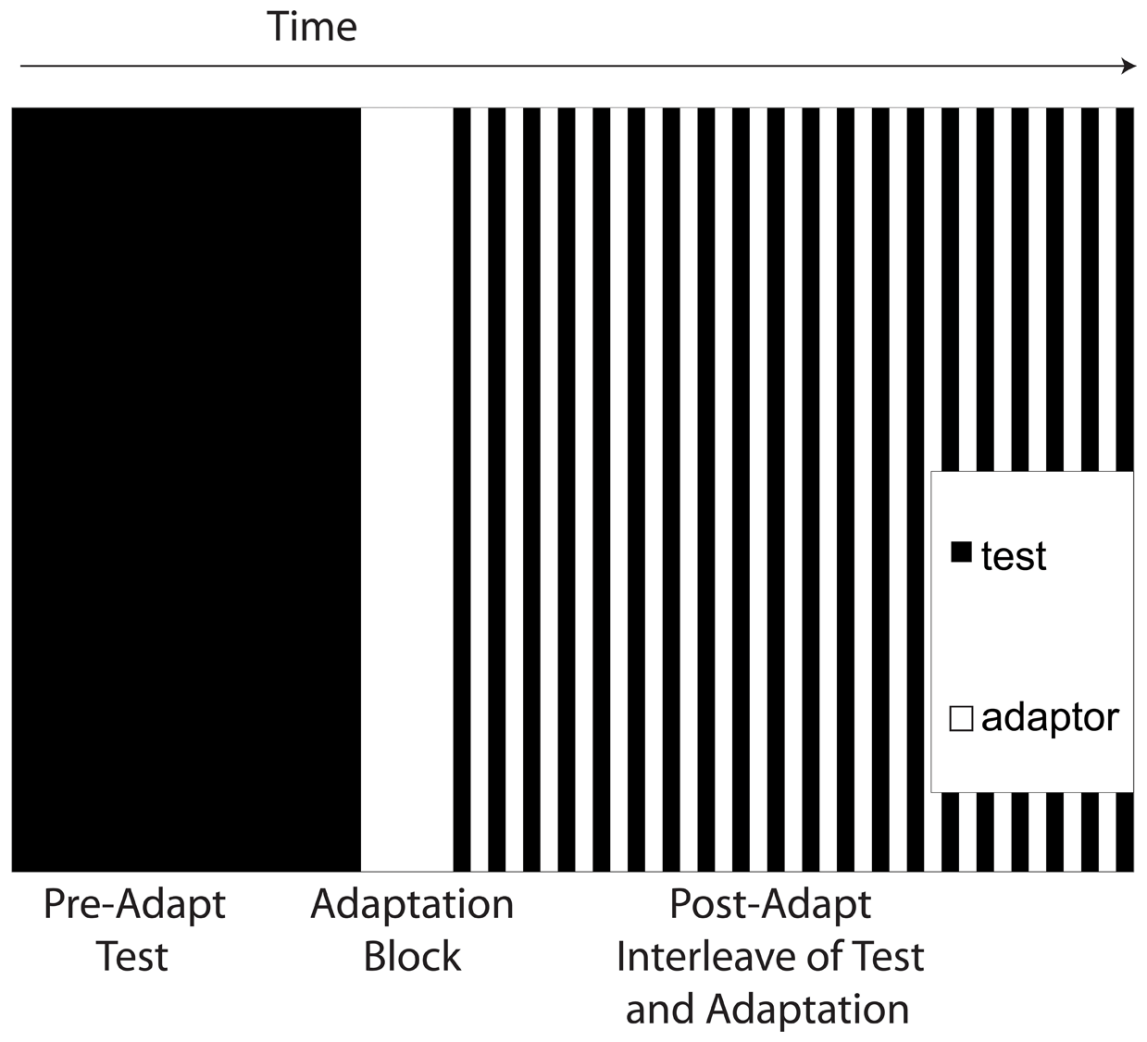
Session structure. Each session began with an initial pre-adaptation block of 140 trials. Participants then experienced an adaptation block of 30 trials, followed by alternating adaptation and test blocks of 7 trials each.

During the initial adaptation phase, participants experienced 30 adaptation stimuli consisting of pulse trains with gaps in them. For instance, a participant might experience three pulses, a gap, three more pulses, another gap, and two pulses. These adaptation pulse trains were presented at an adaptation frequency that was outside of the range of the test frequencies (3 or 5 Hz in the main experiment, and 5, 8, or 12 Hz in the channels experiment). In the main experiment, the gaps were 0.5 seconds for the 3 Hz adaptor and 0.3 seconds for the 5 Hz adaptor. In the channels experiment, the gaps were 1.3–2.3 seconds. Participants reported the number of gaps they experienced on each trial (range of 0–3) and the next trial would begin after the response; most participants took 10–15 minutes to complete the adaptation phase. This adaptation task was designed to require participants to attend to the rhythm of the pulse train (in order to assess whether gaps occurred), but be distinct from the test task. Following the initial adaptation block of 30 trials, additional adaptation and test trials (without feedback) were presented in 20 alternating blocks of 7 trials each. Figure 4 shows the structure of the experimental sessions. On each alternation, the green LEDs would flash and the speaker would play the 440 Hz tone for a longer duration to indicate to the participant that the task was changing.

## Supplementary Material

Supplementary Information Supplemental Note

Supplementary Information Supplemental Table 1
