# Supplementary material for: Rate perception adapts across the senses: evidence for a unified timing mechanism
Source: Sci Rep. 2015 Mar 9;5:8857. doi: 10.1038/srep08857 (PMC4894401; doi:10.1038/srep08857)
Supplement: Supplementary Information — Supplemental Note [file srep08857-s1.pdf]

Supplemental Note:

***Rate perception adapts across the senses: evidence for a unified timing mechanism* by Levitan, Ban, Stiles, and Shimojo**

Maximum-likelihood estimation (MLE) and Bayesian approaches based on reliability have been the dominant computational model for multisensory sensory interactions. In our study, we considered the role of reliability in two contexts: (1) unimodal reliabilities as a predictor of bimodal reliabilities, and (2) unimodal reliabilities as a predictor of the magnitude of adaptation. In both cases, we found deviations from these predictions, suggesting that a substantial modification or extension of those models may be needed to accommodate our results.

When designing our experiment, we initially assumed that the bimodal reliability would be better than the unimodal reliabilities, as predicted by MLE, as we presented no conflicts between auditory and visual rates. Supplemental Figure 1 shows the reliabilities (pooled across all participants) for auditory-only, visual-only, and bimodal conditions as well as the predicted reliability based on MLE. We observed that audition was more reliable than vision for this task, but the bimodal reliability was similar to the auditory-only reliability, rather than the predicted reliability based on MLE. We thus probed this result by pooling pre-adaptation conditions within each participant and conducting a one-way ANOVA on the visual-only, auditory-only, bimodal, and MLE-predicted reliabilities. This ANOVA was significant, $F(3,28) = 15.01$, $p < 0.0001$. Post-hoc comparisons of the bimodal and MLE-predicted reliabilities revealed a significant difference, with the bimodal reliability lower than the MLE-predicted reliability, $t(7) = 5.28$, $p = 0.0011$, and a significant difference between auditory and visual reliabilities, such that auditory reliability was better than the visual reliability, $t(7) = 6.08$, $p < 0.0005$. However, the

bimodal reliability was not significantly different from the auditory reliability, $t(7) = 0.47$, $p = 0.65$. This discrepancy from predictions of MLE is consistent with other work that found that bimodal reliability was better than visual, but not auditory reliability for rate discrimination[1]. However, because our experiment was optimized to measure PSE, as opposed to reliability, we regard these results as suggestive, rather than conclusive. As this was an adaptation study, we chose to use the method of single stimuli and to present the same test values of rate in all conditions. But these choices were not ideal for determining slope. The method of single stimuli creates an internalized criterion, which has some degree of noise. If the magnitude of this criterion noise is large enough, this could lead to apparent violations of MLE. An experiment focused on reliability could instead use a two-alternative forced choice task to better measure reliability and could include more trials. Moreover, the test values could be selected to sample the points on the psychometric function likely to yield precise estimates of the slope. Past experiments in the temporal domain that have been explicitly designed to test MLE predictions for temporal localization of audiovisual stimulation have provided mixed results; there is some evidence "roughly consistent" with MLE integration[2] , but there is also evidence that bimodal precision is not better than unimodal precision[3].

We also considered whether unimodal reliabilities might predict the magnitude of adaptation in the crossmodal conditions. Prominent models of multisensory interactions typically deal with concurrent conflict situations. In such situations, the modality appropriateness hypothesis suggests that more attention will be directed to the more accurate and precise modality, leading to an asymmetry such that the more “appropriate” modality will bias the less reliable one to a greater extent than the converse[4]. A variation of this idea is to recalibrate the modalities proportionally to their reliability; the ratio of those reliabilities has previously been shown to

predict the degree of recalibration for visual-haptic adaptation with concurrent stimulation[5]. A Bayesian model based on reliability was successfully applied to rate perception under conditions of concurrent discrepancy[6]. As we found that audition was more reliable than vision for our task, one might expect that there would be more adaptation in the AV (auditory adaptation, visual test) condition than in the VA (visual adaptation, auditory test) condition, but there was no evidence for this in our data (Figure 2). A straightforward Bayesian model in which adaptation is carried out by a change in the prior would lead to a positive aftereffect, as repeated presentations of a particular stimulus would change the prior so that stimuli similar to the adaptation stimuli would be more likely to be perceived[7], but we found a negative aftereffect. One can construct a more complex Bayesian model that results in a negative aftereffect by taking into account the means and standard deviations of both the prior and the likelihood[8]. But even for concurrent multisensory stimulation, there is current debate about whether reliability predicts the nature of the recalibration resulting from repeated exposure to discrepant stimuli[9,10]; accuracy of each sensory cue, as well as priors about the consistency of mapping between particular sensory cues, may be the critical factors for predicting recalibration[11] in those cue-conflict situations. However, in our experiments there is no sensory discrepancy to resolve. Thus, it is questionable to what extent any of these approaches should be expected to apply; the aftereffect resulting from non-concurrent rate adaptation may reflect a different fundamental mechanism that warrants a different computational model.

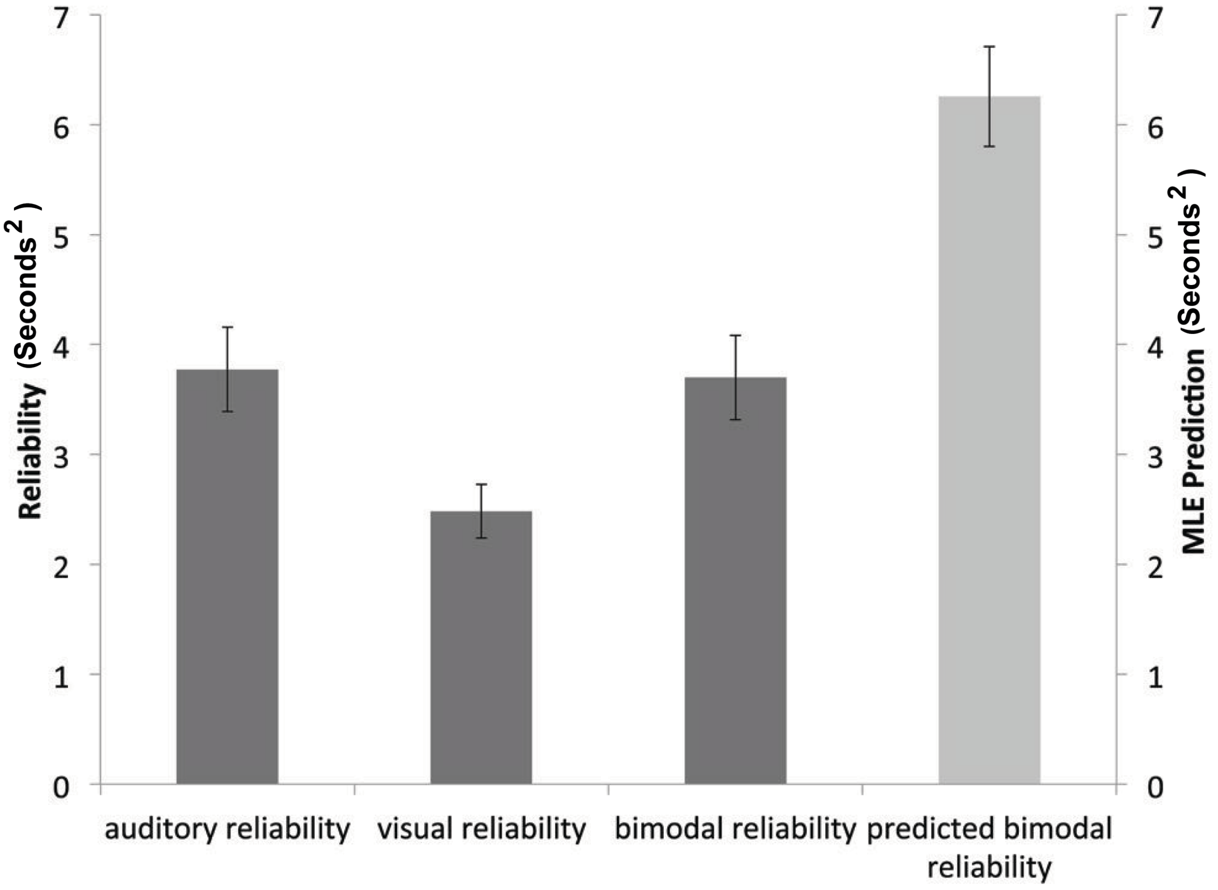

Supplemental Figure 1. Auditory, visual, and audiovisual reliabilities. Pre-adaptation trials in the main experiment were grouped together based on modality (combined across participants), and reliabilities were calculated for auditory, visual, and bimodal audiovisual performance on the rate-classification task (dark bars). Error bars show 95% confidence intervals. The lighter bar depicts the MLE predictions for bimodal audiovisual reliability based upon the unimodal A and V reliabilities, with the error bar representing the 95% confidence interval around this prediction. The audiovisual reliability was not consistent with the MLE predictions.

References:

1 Recanzone, G. H. Auditory influences on visual temporal rate perception. *J. Neurophysiol.* **89**, 1078-1093 (2003).

2 Burr, D., Banks, M. S. & Morrone, M. C. Auditory dominance over vision in the perception of interval duration. *Exp. Brain Res.* **198**, 49-57 (2009).

3 Hartcher-O'Brien, J. & Alais, D. Temporal ventriloquism in a purely temporal context. *J. Exp. Psychol. Hum. Percept. Perform.* **37**, 1383 (2011).

4 Welch, R. B., DuttonHurt, L. D. & Warren, D. H. Contributions of audition and vision to temporal rate perception. *Attention, Perception, & Psychophysics* **39**, 294-300 (1986).

5 Burge, J., Girshick, A. R. & Banks, M. S. Visual-Haptic Adaptation Is Determined by Relative Reliability. *The Journal of Neuroscience* **30**, 7714-7721 (2010).

6 Roach, N. W., Heron, J. & McGraw, P. V. Resolving multisensory conflict: a strategy for balancing the costs and benefits of audio-visual integration. *Proceedings of the Royal Society B: Biological Sciences* **273**, 2159-2168 (2006).

7 Yamamoto, S., Miyazaki, M., Iwano, T. & Kitazawa, S. Bayesian calibration of simultaneity in audiovisual temporal order judgments. *PLoS One* **7**, e40379 (2012).

8 Sato, Y. & Aihara, K. A bayesian model of sensory adaptation. *PLoS One* **6**, e19377 (2011).

9 Di Luca, M., Machulla, T. K. & Ernst, M. O. Recalibration of multisensory simultaneity: Cross-modal transfer coincides with a change in perceptual latency. *Journal of Vision* **9** (2009).

10 Zaidel, A., Turner, A. H. & Angelaki, D. E. Multisensory calibration is independent of cue reliability. *The Journal of Neuroscience* **31**, 13949-13962 (2011).

11 Ernst, M. O. & Di Luca, M. Multisensory perception: from integration to remapping. *Sensory cue integration* [Trommershauser J., Kording K., Landy M. S., eds.] [224-250] (Oxford University Press, New York, (2011).
